# Spatio-temporal evolution and influencing factors of scientific and technological innovation level: A multidimensional proximity perspective

**DOI:** 10.3389/fpsyg.2022.920033

**Published:** 2022-08-11

**Authors:** Yongzhe Yan, Lei Jiang, Xiang He, Yue Hu, Jialin Li

**Affiliations:** ^1^College of Science & Technology, Ningbo University, Ningbo, China; ^2^Business School, Ningbo University, Ningbo, China; ^3^School of Geography and Remote Sensing, Guangzhou University, Guangzhou, China; ^4^Zhejiang Study Base of Individuals of Non-Public Sectors of the Economy, Ningbo University, Ningbo, China; ^5^Department of Geography & Spatial Information Techniques, Ningbo University, Ningbo, China; ^6^Donghai Academy, Ningbo University, Ningbo, China

**Keywords:** multidimensional proximity, scientific and technological innovation, knowledge base, spatial panel econometric model, the Yangtze River Delta region of China

## Abstract

Through a literature analysis, this study proposes that the difference between scientific innovation and technological innovation has been ignored in the current research on the level of scientific and technological innovation and its influencing factors. Combined with multidimensional proximity and knowledge type of current research, a theoretical induction has been carried on their corresponding relation with scientific innovation and technological innovation, research hypotheses were proposed the multidimensional proximity effect on the mode and degree of scientific innovation and technological innovation, five theoretical factors, which are the economic development level, regional economic structure, the level of opening to the outside world, science and technology input and education input, are proposed to affect the level of scientific innovation and technological innovation. In this study, the Yangtze River Delta region of China from 2001 to 2018 is selected as the research sample, and the two hypotheses proposed are tested through a mixed method of exploratory spatial data analysis and spatial panel econometric model. The main conclusions are as follows: i) As an exogenous variable, geographical proximity has a small impact on the level of scientific innovation, but a large impact on the level of technological innovation; ii) As endogenous variables, theoretical influencing factors may not play a significant role in the actual environment due to the complex influence of multidimensional proximity; iii) Based on the idea of improving multidimensional proximity and the actual situation of the region and the city, we can formulate policies conducive to improving the regional and urban innovation environment.

## Introduction

Science and Technology (S&T) are often regarded as the same concept. Many related studies in the academic circle also use a single index to represent the level of science and technology without distinction. But in essence, science and technology are two concepts that are both different and related. Science is man's understanding of the facts and laws of the objective world, mainly based on the causal inference derived from the formal logic system of Greek philosophy and causal relationships by systematic experimental method (during the Renaissance), and used to answer “know-what” and “know-why”. Technology is a means by which man can influence and transform nature and society to solve the problem of “know-how”. It follows that the term “scientific and technological innovation” (STI) can be correspondingly divided into scientific innovation (SI) and technological innovation (TI), which are related and differentiated.

Their relationship is as follows: scientific innovation is to acquire new knowledge, namely a new understanding of the law of the objective world, and new knowledge is often the forerunner of technological innovation. Only through technological innovation can the value of new knowledge obtained from scientific innovation be reflected. Therefore, technological innovation is the bridge to realizing the value of scientific innovation. Currently, relevant studies have attempted to reveal such a relationship between them (Shen et al., 2020). On the other hand, SI is usually unforeseeable while TI has a certain predictability, the reason is that SI aims at understanding and exploring objective laws, but the discovery of such objective laws itself has its unpredictability. The specific development path of scientific discoveries, the timing, and location of breakthrough discoveries, etc., are generally difficult to foresee (Perrons et al., 2021). While TI is a new method or new means adopted to solve practical problems, for its clear purpose, adequate scientific knowledge base, and the corresponding human, material, and financial arrangements, it has certain predictability. Through the division of the concept of STI, we notice that many existing related studies mainly focus on the patent as the proxy variable of innovation output (Costantini et al., 2017; Du et al., 2019), therefore, they essentially measure TI and leave out SI.

Of course, some studies help to differentiate between SI and TI. Most of them choose different proxy variables of S&T for measurements (Park and Suh, 2013; Wong et al., 2014; Fan et al., 2017), which helped to understand this study. However, these studies also make us realize that knowledge, as the basic element of STI, not only has different categories but also plays different roles in STI. Innovation can be regarded as the recombination and connection of different types of knowledge (Schumpeter, 1934; Howells, 2012). Therefore, the research on SI and TI should be based on different types of knowledge. On the contrary, if the fundamental role of different types of knowledge in innovation is not properly defined, the research on innovation may go astray (Howells, 2012). Nevertheless, related works in this domain are still not enough in terms of our knowledge scope.

As the basic element of STI, the research on Knowledge Base (KB) gradually emerged after 2000. Asheim and Coenen (2005) divided knowledge into two categories of knowledge bases: analytical and synthetic KB. Later, it was further differentiated into three categories: analytical, synthetic, and symbolic KB (Asheim, 2007). Classification of KB types is considered to be closely related to innovation types, which in turn is related to the development of enterprises (Grillitsch et al., 2019). Some studies have also pointed out that the structure of regional KB will dynamically affect regional innovation performance, that is, in the long run, regions with three types of knowledge balance are more likely to become innovation leaders (Kveton and Kadlec, 2018). As for the sources of knowledge, Moodysson (2008) points out that there are two approaches to knowledge creation: Global pipeline and Local buzz, which, respectively, apply to analytical KB and synthetic KB. Howells (2012) also emphasizes the individuality and localness of knowledge to demonstrate that knowledge can only grow through communication. Obviously, his statement is more in line with the characteristics of synthetic KB. Martin (2012) described the characteristics of different KB in detail and proposed a method to assess regional KB. In 2013, he noted that knowledge exchange of local configuration is particularly important for industries that rely on symbolic or synthetic KB, while analytical knowledge “flows occur foremost in globally configured networks” (Martin and Moodysson, 2013). In addition, the proposal of the geographical proximity paradox not only deepens the understanding of the impact of geographical proximity (Boschma, 2005) but also promotes the study of knowledge acquisition under various spatial scales (Micek, 2019).

It can be concluded from the above that a considerable part of the research in this field is carried out from the perspective of geographical proximity. In recent years, the research topics on the relationship between geographical proximity and innovation also tend to be more diversified. Examples, new product development (Hong and Lee, 2020), green innovation (Hu et al., 2021), innovation model (Santner, 2018), innovation process (Tanner, 2018), innovation performance (Xiao et al., 2021), and contribution to innovation projects (Santamaria et al., 2021). Importantly, geography is not the only dimension. Boschma (2005) has long proposed that the meaning of proximity not only refers to geographical dimensions actually, but also includes cognitive, organizational, social, and institutional dimensions. Knoben and Oerlemans (2006) summarized three dimensions of proximity, which are geographical, organizational, and technological proximity, and the relationship between multidimensional proximity and knowledge sharing is analyzed. Recent research even involves personal proximity (Leszczynska and Khachlouf, 2018) and network proximity (Yuan and Han, 2021; Zhou et al., 2021). All in all, the academic research on proximity has already broken through the geographical dimension and extended to multiple dimensions (Godart, 2015; Liu et al., 2021; Xiao et al., 2021).

In recent years, studies have become increasingly diversified, some of which have deepened understanding of the role of proximity. For example, the study of Doloreux et al. (2020) revealed that geographical proximity will affect the interaction modes, and then affect the innovation modes. Bennat and Sternberg (2020) pointed out that different knowledge types have different policy requirements. Kapetaniou and Lee (2019) emphasize the importance of geographical proximity even in the open innovation model. In addition, new progress has been made in empirical research on the proximity paradox. Broekel and Boschma (2011) verified that cognitive proximity should be moderate, and too close cognition would hinder innovation performance. Mi et al. (2021) also empirically prove that the importance of related knowledge and unrelated knowledge to innovation can be changed, thus indirectly proving the paradox of cognitive proximity. With the continuous outbreak of COVID-19 across the globe in 2020, more thoughts have been aroused on virtual collaborative research under distanced conditions (Asante et al., 2021). Under the premise of increasing geographical distance caused by isolation measures in many fields such as the economy and society of various countries, it is undoubtedly more significant to study the impact of proximity from multiple dimensions on STI.

In short, the dimensions of proximity have been expanded to include cognition, organization, society, system, culture, and technology in addition to geography, forming a large number of in-depth insights into knowledge and innovation from the perspective of multidimensional proximity. A large part of research in this field has also focused on terms such as collaboration networks and clusters and conducted theoretical and empirical analyses on their role and significance in STI (Ma et al., 2018; Mobedi and Tanyeri, 2019; Dyba et al., 2020; Neulandtner and Scherngell, 2020; Speldekamp et al., 2020). However, this part also emphasizes the difference and connection between geographical proximity and other dimensional proximity in essence, so it is logically consistent with the research on the influence of multidimensional proximity on different types of knowledge. A similar view was declaimed by Boschma (2005) when he proposed the concept of proximity from different dimensions.

Through the review of relevant literature, this study considers that they follow a logical main line of “STI-KB-Proximity”, which agree with a logical dark line of “innovation content-essential characteristics-influencing factors”. Of course, some existing studies also suggest that there are still many knowledge gaps to be bridged in this field (Castellani and Lavoratori, 2020; Chen and Hassink, 2020). Following the logical framework of “STI-KB-Proximity”, we summarize the unclarified themes in this field into the following three points:

(1) Knowledge is regarded as the basic element of STI. However, there are some differences between scientific innovation and technological innovation, and the difference in corresponding knowledge types is not clear in the existing literature. (2) Current studies show that proximity of different dimensions has different impacts on different knowledge types, but the impacts of proximity on STI have not been clarified, and the impacts of the proximity of different dimensions on STI have not been concluded. (3) Empirical studies that attempt to analyze multidimensional proximity and STI are scarce and need to be further expanded.

For this reason, this study chooses the logical dark line of “innovation contents-essential characters-influencing factors” in existing studies, that is, this study will be based on different kinds of KB (hereinafter referred to as the “knowledge”), try to induce their corresponding relation with scientific innovation and technological innovation theoretically, and discuss the influence of multidimensional proximity on the level of scientific innovation and technological innovation, put forward the corresponding research hypothesis, and finally, China's Yangtze River Delta (YRD) region is taken as the research sample, this study describes the spatio-temporal evolution of YRD's SI and TI level, and empirically testifies the research hypothesis combined with other influencing factors.

## Definition of core concepts and research methods

### Definition of core concepts

#### Correspondence and measurement between STI and knowledge types

Combining existing studies (Asheim and Coenen, 2005; Aslesen and Freel, 2012; Martin, 2012; Asheim et al., 2017), this study sets the corresponding knowledge of STI into three kinds of KB: analytical, synthetic, and symbolic knowledge. Analytical knowledge is produced by the scientific method, which is universal, highly abstract, and typified to a large extent. It is common in universities and research institutions. Its innovation form is mainly scientific discovery. Synthetic knowledge is obtained through the application or combination of existing knowledge, which usually exists in the industrial environment and is acquired in the process of interactive learning with customers or suppliers. It has strong recessive and scene-based characteristics, and only part of it can be used to be abstract, summarized, and compiled. Its innovation form is mainly the modification of existing products and processes. Symbolic knowledge depends on the creative process in the project team, which is invisible and highly context-specific. It mainly exists in the process of creating the aesthetic attributes, design, and image of the product, through interaction with the relevant professional team. Its innovation form mainly involves intellectual property works (such as advertising ideas, images, music, video, etc.) (Asheim and Coenen, 2005; Asheim, 2007; Asheim et al., 2017). Through the division of the characteristics of SI and TI in the Introduction, this study proposes that SI mainly corresponds to analytical knowledge, which can be measured by scientific publications such as research articles and research reports. While TI mainly corresponds to synthetic knowledge and symbolic knowledge, which can be measured by patents. Their corresponding knowledge types, main characteristics, and measurement indexes are shown in Table 1.

**Table 1:** Corresponding relationship between STI, knowledge type, and measurement index.

**Innovation type**	**Knowledge type**	**Characteristics and source**	**Innovation entity**	**Measurement index**
Scientific innovation	Analytical knowledge	Easy to encode and spread over long distances. Can be obtained through publications and other knowledge carriers.	Universities, research institutes, companies	Articles, research reports
Technological innovation	Synthetic knowledge	Difficult to record completely, suitable for face-to-face transmission. Acquired through the movement of people with skills.	Universities, research institutes, companies, individuals	Invention patent
	Symbolic knowledge	Hard to record and relies on the local buzz for transmission. Can be obtained through direct interaction with the people involved.	Companies, individuals	Utility model patent, appearance patent, registered trademark

Based on relevant studies (Asheim and Coenen, 2005; Asheim, 2007; Martin and Moodysson, 2013; Davids and Frenken, 2018).

#### The influence mode and degree of multidimensional proximity on STI

Proximity originally existed only in the geographical dimension and was used to represent the spatial or physical distance between innovation entities. In the 1990s, with the introduction of other dimensions by the French School of Proximity Dynamics, the concept of multidimensional proximity, which is closely related to knowledge sharing and innovation^1^, gradually came into being (Boschma, 2005). However, up to now, the five dimensions proposed by Boschma (2005) and the three dimensions proposed by Knoben and Oerlemans (2006) have the “proximity paradox” caused by “lock-in”, that is, close enough distance is conducive to innovation, while too close distance is a hindrance to innovation. “Proximity paradox” can be regarded as the logical starting point of “which is more important in depth or breadth of knowledge search” (O'Connor et al., 2021), so it is a proposition worthy of further exploration. In addition, the proximity of different dimensions is not an independent relationship but can affect each other and to some extent substitute each other. For example, geographical proximity can promote cognitive proximity, but when geographical proximity is insufficient, cognitive proximity or relational proximity can partially replace it (Boschma, 2005; Presutti et al., 2019). It can also be a complementary relationship. In some cases, proximity in geographical, organizational, and institutional dimensions is required to facilitate knowledge transfer and innovation (Knoben and Oerlemans, 2006). Besides, different dimensions of proximity influence technological innovation in different ways (Hung et al., 2021). Based on these characteristics, it can be determined that different dimensional proximity is correlated with each other, and the impact of one single dimensional proximity on innovation is also complex, which is difficult to be linear. Therefore, it may not be appropriate to directly take a certain dimension or multiple dimensions as endogenous variables to analyze their impact on STI. Under this premise, the core research logic of this study is to regard multidimensional proximity as an exogenous variable affecting STI. In other words, SI and TI are dependent variables, and their independent variables need to be determined in other ways, while multidimensional proximity is a preset constant that may affect independent variables and dependent variables.

Multidimensional proximity has an impact on different knowledge types, while different influence degree is generally assured by scholars (Davids and Frenken, 2018). Geographical proximity is conducive to interactive learning and promotes tacit knowledge learning (Boschma, 2005; Knoben and Oerlemans, 2006). A recent study proved that geographical proximity at the level of hundred meters is conducive to knowledge transferring among SMEs, and the distance beyond this level declines rapidly (Rammer et al., 2020). So the influence degree on synthetic knowledge and symbolic knowledge should be higher. Analytical knowledge is a kind of knowledge that is easy to encode and can be transmitted remotely. Even if geographical proximity is insufficient, it can be made up of cognitive, organizational, or institutional proximity, which is not enough to affect knowledge absorption and innovation (Davids and Frenken, 2018). According to the corresponding relationship in Table 1, synthetic knowledge and symbolic knowledge correspond to TI, and analytical knowledge corresponds to SI. Therefore, this study proposes research hypothesis (1):

Hypothesis (1): Geographical proximity has a greater impact on the TI level than on the SI level.

### Research methods

#### Exploratory spatial data analysis (ESDA)

ESDA is a common method in economic geography research, which is used to analyze whether there is a correlation in the spatial distribution of a geographical element, the significance of correlation degree, and the measurement of size. Spatial autocorrelation analysis can be divided into global spatial correlation analysis and local spatial correlation analysis, which can be represented by global Moran's I and local Moran's I, respectively. The calculation formula of global Moran's I is as follows:


(1) $$I=\frac{n}{W}\frac{\sum_{i=1}^{n}\sum_{j=1}^{n}w_{ij}(x_{i}-\bar{x})(x_{j}-\bar{x})}{{\sum_{i=1}^{n}\left(x_{i}-\bar{x}\right)}^{2}}$$

In formula (1), n is the total number of regions in the study area, w_ij_ is the spatial weight, W is the sum of the spatial weights, x_i_ and x_j_ are the attributes of region i and region j, respectively. $\bar{x}$ is the average value of attributes. According to the corresponding relationship shown in Table 1, this study uses global Moran's I to conduct spatial autocorrelation analysis on the corresponding indexes of SI level and TI level of cities in the YRD region.

#### Spatial panel econometric model

Classical econometric models cannot estimate the possible spatial dependence between independent variables and dependent variables. The spatial dependence means that the attributes of similar units in geographical space will influence each other and tend to be close or the same. One of the objectives of this study is to determine the impact of geographical proximity on scientific and technological innovation. Consequently, spatial dependence is an essential factor in our model construction. Paelinck and Klaassen (1979) first proposed the concept of the spatial econometric model, Cliff and Ord (1981) put forward Spatial Autoregressive Model. Since Anselin (1988b) systematically organized the concepts, methods, and models of spatial metrology, it has been continuously developed and practiced in many disciplines. Spatial econometric models are a collection of techniques for dealing with special problems caused by space in statistical analysis models of regional science (Anselin, 1988b). However, early models were built around cross-section data. Since 2000, studies on spatial panel econometric models have been emerging (Elhorst, 2003; Baltagi, 2005; Kapoor et al., 2007), becoming a new important direction in this field. The spatial panel econometric model is an extension of the traditional panel model, which is formed by incorporating the spatial effects of regional or sectional dimensions. Among them, the spatial effects between dependent variables, independent variables, and residuals are called endogenous spatial effects, exogenous spatial effects, and interaction effects in the residuals respectively, and the spatial panel model can be divided into seven categories according to the above three effects and their combination. The first three basic types were the Spatial Lag Model (SLM) or Spatial Autoregressive Model (SAR), Spatial Lag of X Model (SLX), and Spatial Error Model (SEM), which only contain endogenous spatial effects, exogenous spatial effects, and interaction effects in the residuals, respectively. The last four models contain more than two kinds of spatial effects, those Spatial Durbin Model (SDM), Spatial Durbin Error Model (SDEM), Kelejian and Prucha Model or SAC Model, and General Nesting Spatial Model (GNSM). The Spatial Dynamic Panel Data Model (SDPD) is formed by adding the dynamic change term of temporal dimension, namely the temporal lags, based on the above Model (Lesage and Pace, 2009). And Elhorst (2012) was the first scholar to study the SDPD in depth.

When studying practical problems, there are usually two ways to choose the above models. One is from the special to the general, that is, starting from the classical panel model, adding spatial lag terms one by one, and finally getting the GNSM. The selection criteria include the Lagrange Multiplier test (LM) (Burridge, 1980; Anselin, 1988a), And the Robust Lagrange Multiplier test (robust-LM) (Anselin et al., 1996). The other is to start from the general to the special, that is, starting from the GNSM, and degenerating it into the specific spatial model by deleting the specific spatial lag term. As advocates of the SDM, Lesage and Pace (2009) believe that the SDM should be the starting point for research, so as to avoid the risk of missing variables. And its general setting form is:


(2) $$\begin{array}{r} y=\rho Wy+X\beta +WX\theta +\varepsilon \end{array}$$

In formula (2), y represents the dependent variable of n by 1 vector. ρ is the spatial autoregressive coefficient to be estimated. W represents the spatial weights matrix of n by n, which is set as a proxy variable of proximity and an exogenous variable of the model. Wy represents the spatial lags of the dependent variable. X is the explanatory variable matrix of n by k.β represents the coefficient of k by 1 vector to be estimated. θ represents the coefficient of spatial lags of k by 1 vector to be estimated. ε is the error terms of n by 1 vector. If ρ = 0, the SDM degenerates into SLX. If θ = 0, the SDM degenerates into SLM. If ρ = θ = 0, the SDM degenerates into the traditional panel model. If ε = λWε+ν, that is, ε contains the spatial autocorrelation error terms, then the SDM evolves into the SDEM. If the temporal lags y_t−1_ and spatial lags of the time lags Wy_t−1_ were added, it forms the SDPD. According to the characteristics and purpose of our research object, this study will choose the appropriate spatial econometric model, and analyze the results of different models by horizontal comparison.

According to the corresponding relationship in Table 1, the total number of studies published in Chinese and foreign languages in the study region was selected as a proxy variable of the local SI level in that year. The total number of domestic patent applications approved in the research region was selected as a proxy variable of the local TI level in that year. The total number of Chinese studies is obtained through the China National Knowledge Infrastructure (CNKI), and the total number of foreign studies is obtained through the Web of Science. The total number of patents approved includes the total number of invention patents, utility model patents, and appearance patents, which are obtained from the China Urban Statistical Yearbook, China Statistical Yearbook, and the official websites of local statistical departments.

## Spatio-temporal characteristics of the level of STI in the Yangtze River Delta region

### Study area

As the largest developing country in the world, China once made remarkable achievements in the history of the ancient S&T in the world but fell behind significantly in modern times. The Needham Question, which was caused by the slow-down of modern China's S&T, has been widely concerned (Lu, 2015). In recent years, China has become one of the focuses of global attention due to its rapid economic and technological development. Located in the lower reaches of China's Yangtze River, the YRD is an alluvial plain formed before the Yangtze River enters the East China sea. It covers an area of 21,700 square kilometers or about 2.2% of China's total land area. In terms of economic development, the GDP of the YRD reached 24.5 trillion yuan in 2020, with a population of 227 million, accounting for 24.2 and 16.1% of China's total. The urbanization rate of the permanent population has exceeded 60%, ranking among the top five urban agglomerations^2^ in China. In terms of scientific and technological levels, we retrieved ESI's highly cited studies published by Clarivate on January 25th, 2022. Of the top 20,000 studies from Mainland China, 7,665 studies were published (or participated in) by research institutions in the YRD region, accounting for 38.3% of the total in China. According to the database of the China Patent Announcement and Announcement System^3^, 2,514,170 patents were issued in the YRD region, accounting for 28.5% of China's total. According to the above indicators, it can be inferred that the SI level and TI level in the YRD region are position-leading in China. Therefore, it is of certain typicality and important research value to take China's YRD region as the research sample region.

**Figure 1:**
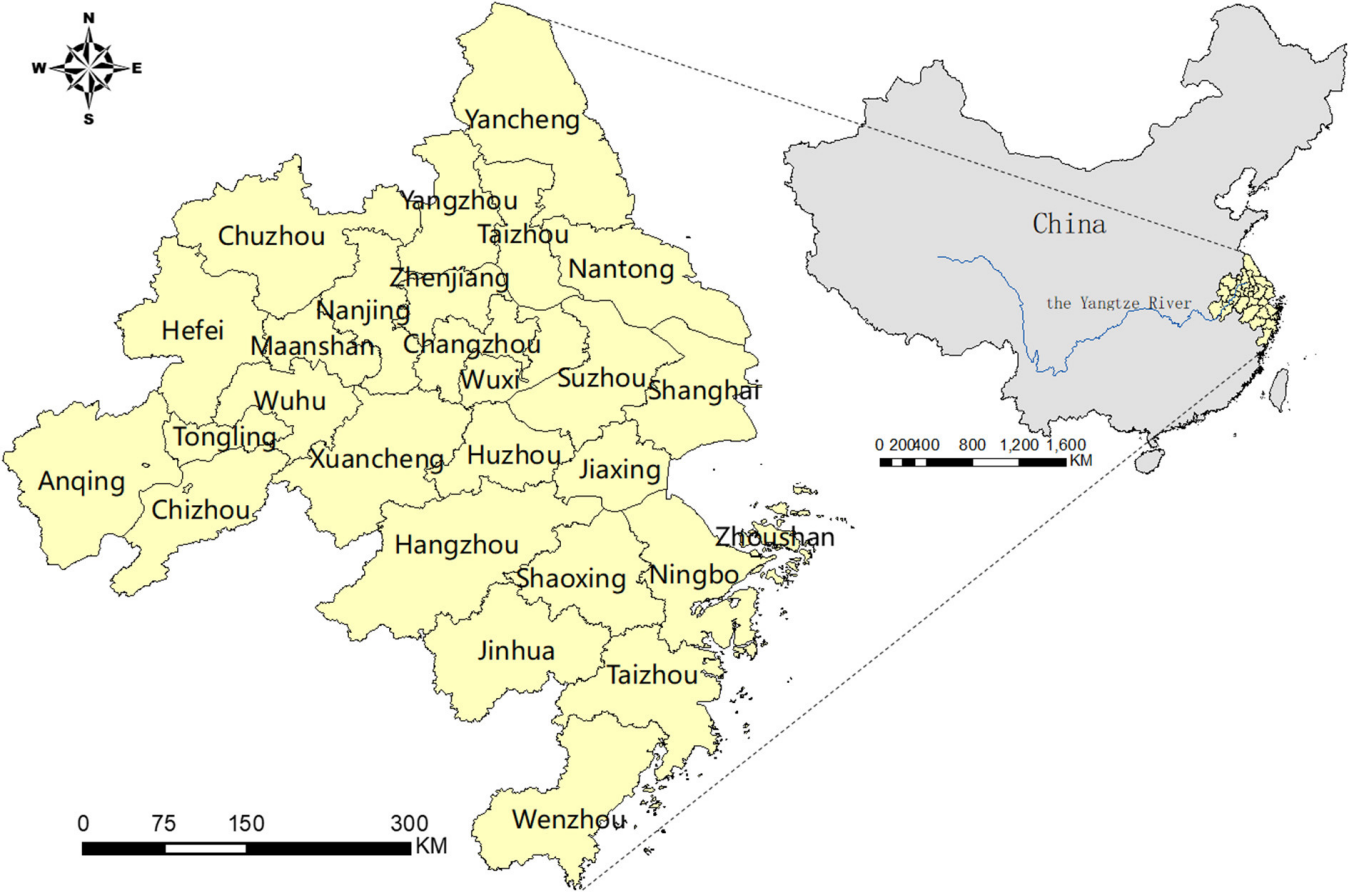
Geographical location distribution of 27 central cities in YRD.

In terms of regional scope, the administrative region of the YRD includes three provinces and one city, namely Jiangsu Province, Zhejiang Province, Anhui Province, and Shanghai City. According to the Outline of the Yangtze River Delta, Regional Integration Development Plan issued by the Chinese State Council in 2019. Twenty seven cities were selected as central regions, those are Shanghai, Nanjing, Suzhou, Wuxi, Changzhou, Zhenjiang, Nantong, Yangzhou, Yancheng, Taizhou in Jiangsu Province, Hangzhou, Ningbo, Wenzhou, Huzhou, Jiaxing, Shaoxing, Jinhua, Zhoushan, Taizhou in Zhejiang Province, Hefei, Wuhu, Maanshan, Tongling, Anqing, Chuzhou, Chizhou, and Xuancheng in Anhui Province. Therefore, this study chooses these 27 cities as the sample regions for research (the location distribution is shown in Figure 1). In terms of the research time range, China joined the WTO in 2001, and the economy of the YRD region began to enter a stage of sustained and rapid growth. In 2018, the “Regional integration of the Yangtze River Delta” was promoted as China's national strategy, marking a new stage of the development of the YRD. Consequently, this study selects corresponding indicators from 2001 to 2018 to analyze the level of STI and its influencing factors.

### Spatio-temporal change of the SI level and TI level

**Figure 2:**
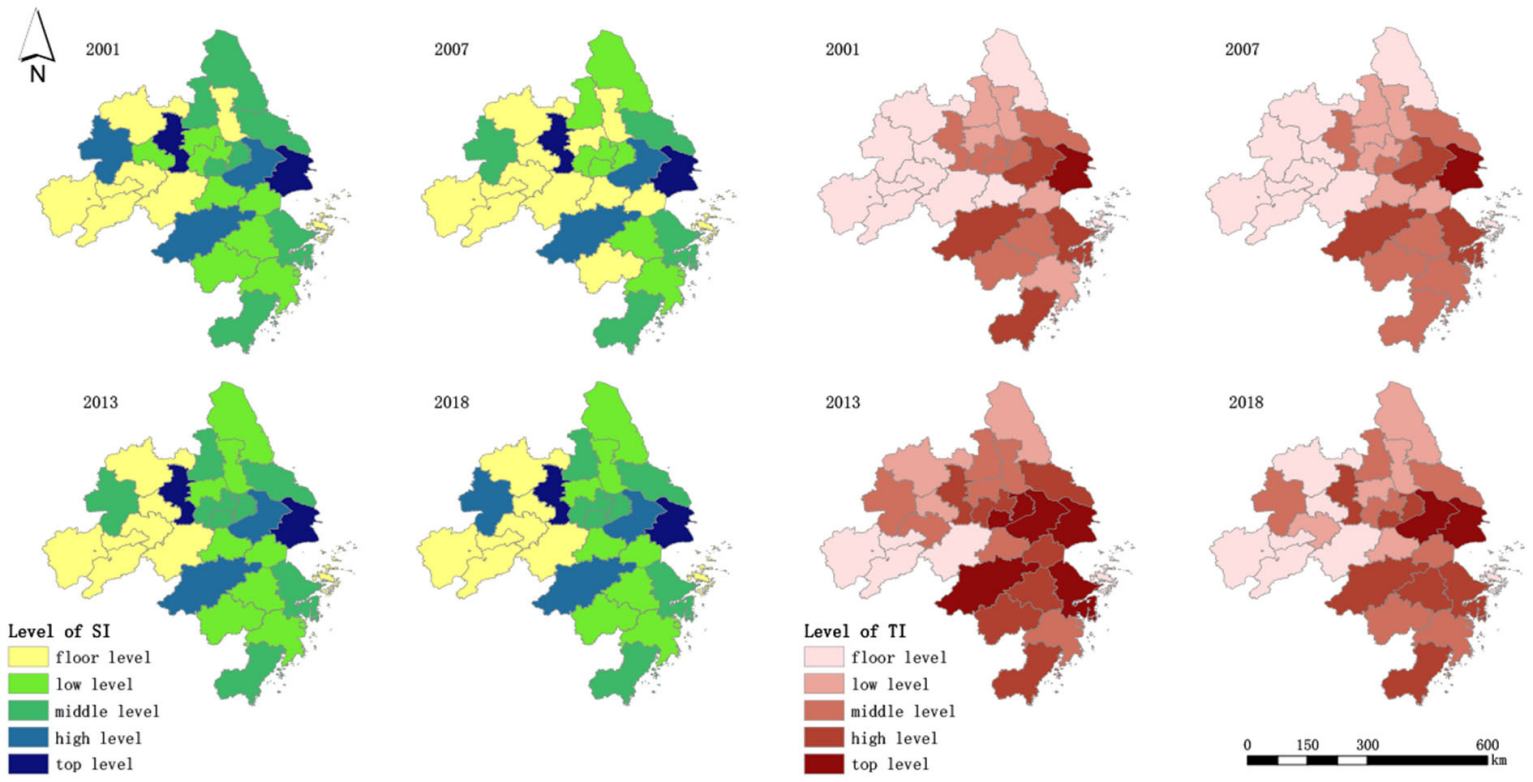
Spatial distribution of the SI level and TI level.

Software ArcGIS 10.5 was used to conduct spatial visualization analysis on the SI and TI levels of 27 central cities in the YRD region from 2001 to 2018 (Figure 2), and the results showed different spatial distribution characteristics. Take 2001, 2007, 2013, and 2018 as an example, the SI level of Shanghai, Nanjing, Hangzhou, and Hefei present a multipolar distribution, but around Shanghai, Suzhou, Nantong, Jiaxing, and Ningbo gradually enhance their SI level, and cities around Hefei has been belong to the low-lying land of SI level. There is no obvious high SI level agglomeration among cities in different years. In terms of TI level, Shanghai generally takes the lead, while neighboring cities such as Suzhou, Hangzhou, and Ningbo rotate to follow Shanghai. Except for cities in southern Zhejiang, Shanghai is the center of TI high-level agglomeration, and a decreasing trend from east to west and from south to north is in all.

### Spatial autocorrelation analysis

Considering the size difference of each city, after the normalization of the total number of studies and the total number of patents in each region, the spatial autocorrelation test of SI level and TI level in the YRD region is carried out by the global Moran's I, and the results still reflect differences between the two. Taking 2001, 2007, 2013, and 2018 as an example (Table 2), the Moran's I of SI level is negative, and the *p* value is not significant, indicating that its performance is mainly spatial random distribution. Besides 2001, the Moran's I of TI level is significant, at least 10% level in the other 3 years, indicating that spatial dependence is dominant. These characteristics preliminarily confirm research hypothesis (1), that is, SI is less affected by geographical proximity, so the main distribution show is geographical spatial random, and TI is greatly affected by geographical proximity, thus showing spatial dependence.

**Table 2:** Moran's I of SI and TI level.

**Innovation type**	**Year**	**Moran's I**	**Z value**	***p* Value**
SI	2001	−0.058	−0.209	0.834
	2007	−0.067	−0.295	0.768
	2013	−0.057	−0.188	0.851
	2018	−0.072	−0.331	0.740
TI	2001	0.061	1.052	0.293
	2007	0.210	2.660	0.008***
	2013	0.177	1.857	0.063*
	2018	0.223	2.198	0.028**

***, **, and * represent the significance level under 1, 5, and 10%, respectively, which are consistent in the following table.

However, some attributes of spatial units show spatial autocorrelation, which may be caused by spatial dependence due to the influence of exogenous variables, spatial interaction of endogenous variables, or statistical errors (Anselin, 1988b). To clarify the reasons for the spatial random distribution of SI level and the spatial dependence of TI level in the YRD region, and further verify the research hypothesis (1). In the next part of this study, possible influencing factors will be selected according to the relevant studies. And the reasons will be analyzed in more detail by selecting the applicable spatial econometric model.

## Analysis of influencing factors of SI and TI level

### Model specification

There is a certain amount of research conducted on production mode and function of knowledge. Typical Knowledge Production Function (KPF), such as The study of Griliches (1986) and Griliches (1992), focuses on the production and spillover of regional knowledge. Jaffe (1989) expanded and improved the knowledge production function proposed by Griliches, forming a paradigm of Griliches–Jaffe knowledge production function. Later, Anselin et al. (2000) applied a spatial econometric model to further expand the knowledge production function, and a study by Fritsch (2002) completely separated intraregional and interregional spillover effects. These researches follow the paradigm of Griliches–Jaffe knowledge production function, which provides great inspiration for this study. However, since SI and TI are distinguished in this study, the corresponding knowledge types should also be distinguished from each other to construct an independent model. To avoid the Scale Economy Effect of different power assignments on functions, we chose the general linear regression model for coefficient estimation, focusing on clarifying the respective roles of exogenous and endogenous variables in this study.

There have been many studies on influencing factors of the STI level (Bronzini and Piselli, 2016; Howell, 2017; Piperopoulos et al., 2018; Khattak et al., 2020; Razzaq et al., 2021), based on the definition of SI and TI in this study, we select five factors that theoretically affect both of them: economic development level, regional economic structure, degree of opening up, expenditure in S&T, and expenditure in education. The corresponding proxy variables are local GDP, the proportion of output value of the tertiary industry in GDP, the amount of foreign capital actually utilized, the S&T expenditure, and the education expenditure in the local financial budget. Panel data of five indicators are obtained by collecting the corresponding index data of 27 cities in the YRD from 2001 to 2018 (Table 3). According to the research conclusions of relevant literature, this study puts forward the research hypothesis (2):

Hypothesis (2): As endogenous variables, the economic development level, regional economic structure, degree of opening up, expenditure on S&T, and expenditure on education all have a positive impact on SI level and TI level.

**Table 3:** Descriptive statistical results of panel data of influencing factors.

**Variables**	**Symbol**	**Obs**.	**Mean**	**Std. Err**.	**Min**.	**Max**.
Economic development level	GDP (100 million CNY)	486	3,267.9	4,261.7	61.9	32,679.9
Regional economic structure	IND (%)	486	41.1	8.0	23.4	69.9
Degree of opening up	FDI (10 thousand USD)	486	175,545.5	268,908.1	334	1,851,378
Expenditure in S&T	SCE (10 thousand CNY)	486	176,572.2	447,500.6	20	4,263,655
Expenditure in education	EDU (10 thousand CNY)	486	663,285.9	1,087,174	4,274	9,179,869

In terms of model selection, we followed Lesage and Pace (2009)'s advice and took SDM as the starting point of analysis. Meanwhile, we adjusted the model according to the characteristics of our research objects and research needs and made a horizontal comparison between the analysis results of different models to determine the most explanatory selection.

The selection of spatial weights matrix is the core and key part of the spatial econometric model. While the selection criteria depend on the research object and leave no unified standard (Jiang, 2020). This study argues that multidimensional proximity can be regarded as exogenous variables to construct different spatial weights matrices and conduct spatial econometric model analysis. However, due to the rich connotations of these dimensions, it is not a unified proposition for constructing an accurate and reasonable spatial weights matrix in the academic circle today either (Lesage and Pace, 2009). Because multidimensional proximity is usually time-varying, the corresponding spatial weight matrix should also be dynamic. In addition, there is a correlation between different dimensional proximity, so the constructed spatial weights matrix also has theoretical interaction. Accordingly, it is necessary to select a more complex spatial econometric model of a high-order spatial weights matrix, which will be a challenge beyond the scope of this study. Therefore, we choose the geo-spatial weight matrix with clear meaning and no change over time to represent the influence of geographical proximity as an exogenous variable. Due to the relatively perfect transportation infrastructure in the YRD region, the time distance differences between major central cities are all at the level of hours. Therefore, the spatial weights matrix based on Queen and the K-nearest neighbors are selected. Finally, the influencing factors analysis model of SI level and TI level is constructed as follows:


(3) $$\begin{array}{r} Y_{jit}=\rho_{j}WY_{it}+X_{it}\beta_{j}+WX_{it}\theta_{j}+\varepsilon_{j} \end{array}$$

In formula (3), Y_jit_ represents the total number of study (if j = 1) or the total number of patents (if j = 2) that City i owns in the year t, respectively, to represent its SI level or TI level. X_it_ is a vector of independent variables; ρ_j_ represents the unknown spatial lag coefficient of the dependent variable to be estimated. β_j_ represents the unknown coefficient vector of the independent variable to be estimated. θ_j_ represents the coefficient vector of the unknown spatial lags term of the independent variable to be estimated. ε_j_ represents the spatial error terms. W represents the spatial weights matrix, i.e., the exogenous variables preset according to geographical proximity. j = 1 or 2.i = 1, 2, …, 27. t = 1,2, …, 18.

### Influencing factors of SI level in the YRD region

Because SI is corresponding to analytical knowledge, according to the related research, it can be based on the influence from multiple dimensions, such as social, organizational, institutional, or cognitive proximity, while influence based on geographic proximity is not significant. In consequence, influence from SI levels of the geographic adjacent area is not significant in the local region. However, the influencing factors (Economic development level, Degree of opening up, etc.) of adjacent regions obviously have an impact on the influencing factors of the local region, so this study considers choosing the Spatial Lag of X Model (SLX). However, according to Lesage and Pace (2009)'s suggestion, this study still takes the SDM as the starting point for model setting. Through the Hausmann test, it is found that the random effect is better than the fixed effect. At the same time, the spatial autocorrelation analysis also indicates that the spatial random distribution of SI level may be influenced by exogenous variables unrelated to explanatory variables, that is, the proximity of other dimensions except for geographical proximity. Consequently, random effects are selected. Considering that the SI level in the YRD region is distributed randomly in space, we set a traditional non-spatial model (NSM) without spatial effects for comparison, to clarify the difference in the impact after considering the exogenous variable of geographical proximity. The results are shown in Table 4.

**Table 4:** Regression model results of influencing factors of SI level.

**Model**	**SDM**		**NSM**
**Variables**	**Coefficient**	**Total effect**	**Coefficient**
GDP	1.754*** (0.198) 140.340*** (52.344)	1.135*** (0.368)	1.540*** (0.206)
IND		−116.322* (59.551)	-142.045*** (41.984)
FDI	0.005*** (0.002)	0.022*** (0.004)	0.0106*** (0.002)
SCE	−0.0002 (0.002)	0.006** (0.002)	0.005*** (0.002)
EDU	0.002** (0.001)	−0.002 (0.002)	−0.001 (0.001)
Constant	5625.952** (2764.249		7230.757*** (2251.972)
W*GDP	-0.687* (0.366)		
W*IND	-245.558*** (68.743)		
W*FDI	0.0149*** (0.004)		
W*SCE	0.006** (0.002)		
W*EDU	-0.005*** (0.002)		
ρ_1_	0.061 (0.068)		
R^2^	0.745		0.641
Obs.	486		486

The numbers in brackets represent the standard error for the corresponding coefficients.

***, ** and * represent the significance level under 1%, 5% and 10% respectively, which are consistent in the following table.

As shown in Table 4, the spatial autoregression coefficient ρ_1_ of SDM is close to 0 and is not significant, which indicates that SDM can degenerate to SLX^4^. Again, it indicates that there is no spatial autocorrelation between SI levels in YRD regions, which is consistent with the conclusion in Section Spatial autocorrelation analysis that SI levels are randomly distributed in space. To investigate whether the explanatory variables have spatial effects, we can compare the regression results of SDM and NSM. In the results of NSM, the coefficients of GDP, FDI, and SCE are significantly positive, indicating that the economic development level, the degree of opening up, and the expenditure in S&T all have a significant positive impact on SI. However, the coefficients of IND and EDU are negative, which contradicts research hypothesis (2) and needs to be explained by SDM. In the results of SDM, the coefficients IND and EDU are significantly positive, but coefficients of W^*^IND and W^*^EDU are significantly negative and their absolute value is larger, indicating that the local economic structure and educational expenditure still have a significant positive impact, but the economic structure and educational expenditure in neighboring areas have a higher negative impact. In the coefficients of the Total effect caused by superposition, IND is significant at the 10% level but EDU is not. The results indicate that the local regional economic structure still has a positive effect on local SI, but may be affected by the “siphon effect” from neighboring regions, resulting in a negative effect on the final result. In addition, this superposition effect also exists for the coefficients of GDP, FDI, and SCE, among which the positive impact of local GDP on SI is originally larger than the Total effect (1.754 > 1.135), but is pulled down due to the negative impact of neighboring regions on local GDP (W^*^GDP = −0.687). Local FDI was enhanced by the positive influence of FDI from neighboring regions (W^*^FDI = 0.0149). The effect of local SCE was negative but not significant, while the relatively more significant effect from neighboring regions' SCE (W^*^SCE = 0.006) “spilled over to the local region, resulting in a significantly positive final effect (Total effect = 0.006). In conclusion, the SDM model has better goodness of fit than the NSM model (0.745 > 0.641). In terms of the explanation of the influencing factors of SI level in the YRD region, it also distinguishes the different influences from local and neighboring regions more clearly. It also indicates that although the local SI level may not be directly affected by the neighboring regions' SI level, local influencing factors are still affected by the neighboring region, that is, explanatory variables have spatial effects.

### Influencing factors of TI level in the YRD region

TI mainly corresponds to synthetic knowledge and symbolic knowledge. According to research hypothesis (1), it is expected that geographical proximity impacts both significantly dependent and explanatory variables, so SDM is still considered as the starting point for analysis. The Hausmann test shows that the fixed effect is better than the random effect, and the LR test shows that a two-way fixed effect should be adopted. This feature also fits with reality, that is, leading industries in many cities present high aggregation (such as new and high technology industries in Shanghai, Suzhou, and Ningbo manufacturing). Since the main entities of TI, which are represented generally by industries in these cities, not only they have a rich industrial foundation and talent advantage but also technical barriers are formed, which makes it difficult for other cities to copy in the short term. This constitutes an important source of the two-way fixed effect in SDM.

In addition, the applicability of SEM is also considered. The results of Robust LM-error and Robust LM-lag tests show that the SLM should be considered. Consequently, SDM is finally selected. At the same time, considering that regional TI will be affected by past technological basis, SDPD is also used for analysis to judge the dynamic responses under the condition of temporal lags. The results are shown in Table 5.

**Table 5:** Regression model results of influencing factors of TI level.

**Model**	**SDM**		**SDPD**	
**Variables**	**Coefficient**	**Total effect**	**Coefficient**	**Total effect**
	**(Std. Err.)**	**(Std. Err.)**	**(Std. Err.)**	**(Std. Err.)**
GDP	6.3618***	2.8525***	2.2381***	12.2962
	(0.3241)	(0.8498)	(0.3472)	(33.4872)
IND	124.3173	956.1911***	−12.7428	768.4356
	(82.2271)	(151.8691)	(66.4413)	(2402.1280)
FDI	0.0043	−0.0090	0.0022	−0.0113
	(0.0033)	(0.0088)	(0.0027)	(0.1148)
SCE	−0.0026	−0.0024	0.0018	−0.0052
	(0.0024)	(0.0055)	(0.0019)	(0.0977)
EDU	−0.0112***	−8.12E- 06	−0.0057***	−0.0282
	(0.0016)	(0.0033)	(0.0013)	(0.0930)
W*GDP	−4.1304***		−0.9710***	
	(0.6492)		(0.5008)	
W*IND	638.9875***		80.1936	
	(127.0841)		(88.7281)	
W*FDI	−0.0114*		−0.0036	
	(0.0066)		(0.0048)	
W*SCE	0.0006		−0.0019	
	(0.0041)		(0.0030)	
W*EDU	0.0113*		0.0027	
	(0.0027)		(0.0022)	
PAT_t−1_			0.7497***	
			(0.0343)	
W*PAT_t−1_			−0.2924***	
			(0.0628)	
ρ_2_	0.2059***		0.4300***	
R^2^	0.828		0.933	
Obs.	486		459	

Results of the SDM model show that the spatial lag coefficient of TI is significantly positive, indicating that the TI level in adjacent areas has a significant spatial spillover effect on the local area, which is consistent with the statement in hypothesis (1) that TI is more affected by geographical proximity. It is also consistent with the empirical analysis of the spatial autocorrelation of TI. For the coefficient of explanatory variables, the coefficients of GDP and EDU are significant at a 1% level, indicating that among the local influencing factors, only the economic development level and expenditure on education have a significant impact on TI, and the former is positive, while the latter is negative. The positive pulling effect of the former is consistent with the above analysis, that is, the local economic development level also plays a positive impact on TI. One possible explanation for the negative effect of the latter is that China's patent stimulus policy from the central to local governments once promoted a surge in the number of patents while leading to a decline in patent quality (Kai and Rensheng, 2017). Under the premise of policy orientation changes and patent saturation in specific technology fields (Liming and Haibo, 2015), the number of patent grants tend to decline, which was consistent with the fact that the number of patent grants in most cities in the YRD region declined around 2012. So, the negative effect of the latter may be a statistical fallacy^5^ after subtracting the effect of patent incentives. Another possible explanation comes from the model level because the spatial weight matrix selected here is the first-order spatial weight matrix constructed based on the Queen criterion. Academic circles believe that different choices of the spatial econometric model and weight matrix type have a great impact on the significance and size of coefficients^6^. Therefore, negative coefficients may also come from other types of models based on high-order spatial weight matrix. On the other hand, for the coefficient of explanatory variables in neighboring regions, both W^*^GDP and W^*^IND are significant at a 1% level, and the former is negative while the latter is positive. It shows that the economic development level and the Regional economy structure in the neighboring region play a “siphon” and “trickle-down” effect respectively. In terms of the total effect, coefficients of GDP and IND are significantly positive, indicating that the economic development level and Regional economic structure have a relatively significant positive impact on TI, while the coefficients of other influencing factors are not significant.

Results of SDPD show that the spatial lag coefficient (ρ = 0.430), time lag coefficient (PAT_t−1_ = 0.750), and spatiotemporal lag coefficient (W^*^PAT_t−1_ = −0.292) are significant at the 1% level, indicating that the local TI level can be promoted by the TI level of neighboring regions in the current year and the local TI level of its previous year, but the TI level of neighboring regions of previous years is negative. This is possibly conflicts and competition in patent registration protection between cities leading to a “zero-sum game” situation (Yu and Zhang, 2019; Suzuki, 2020; Deuk, 2021). The coefficients of other variables are consistent with SDM or are not significant, so they will not be described again.

### Robustness test and discussion

In above parts of this study, the influencing factors of SI level and TI level in the YRD region are analyzed, respectively. In order to test the robustness of the conclusion, the following attempts are made: the influencing factor data is replaced by per capita data. The spatial weight matrix was replaced by a k-order nearest neighbor matrix and a randomly generated spatial “absurd matrix”, and the results showed that the conclusion was robust. The conclusion is as follows: For the SI level, the local economic development level, the degree of opening up, and the Expenditure in S&T all have a significant positive impact. However, there are positive and negative effects of the corresponding influencing factors in the neighboring regions, and these three influencing factors are still significantly positive after superposition, indicating that they promote the improvement of SI levels in the YRD region. The impact of local regional economic structure and expenditure on education is significantly positive, but the impact of adjacent regional economic structure and expenditure on education is significantly negative and higher in absolute value. After superposition, the former is negative while the latter is not significant, indicating that the improvement of regional economic structure reduces the SI level overall. In addition, the influence of adjacent SI levels on the local SI levels is not significant, which may be because the local SI level is affected by the proximity to other dimensions. For TI, both the local economic development level and the regional economic structure of neighboring regions can promote its improvement, but the economic development level of neighboring regions and local expenditure on education can hinder its improvement. After superposition, the improvement of economic development level and regional economic structure can promote the TI level. The TI level of the neighboring region can promote the local TI level, and for a dynamic response, the local TI level in the previous year can promote the local TI level in the current year.

In the above analysis, through the spatial econometric model with a spatial weight matrix based on geographical position, geographical proximity is proved as exogenous variable, that impacts the influence factors spatial differently, namely influence factors do not all play a role equally in the area of the YRD region, but the existence of spatial heterogeneity. At the same time, it also shows that the SI level is not significantly affected by geographical proximity, which may be because of proximity from other dimensions, but the influencing factors of the SI level are still affected by geographical proximity. Since the TI level and its influencing factors are significantly affected by geographical proximity, the research hypothesis (1) proposed in this study is proved. The improvement of the SI level needs to be expanded from other dimensions such as cognition, institution, society, and organization, and the spatial distribution of these dimensions may not be consistent with geographical spatial distribution. For example, Shanghai's SI may be close-related to Beijing, Tokyo, Oxford, Boston, or other cities, which are distant geographical distances but close cognitive distances. On the other hand, TI is more likely to overflow from the geographical adjacent regions, so the TI level in cities adjacent to Shanghai is also very high.

On the other hand, according to the above analysis results, the research hypothesis (2) has only been partially proved, that is, the five influencing factors that should theoretically promote S&T development have only partially passed the significance test. This may be because our study design was unable to measure the effects of geographical proximity while excluding proximity from other dimensions (Knoben and Oerlemans, 2006). That is, proximity from other dimensions as exogenous variables may completely affect the significance, coefficient size, and even sign plus or minus of the influencing factors as endogenous variables.

In practice, we can combine the characteristics of the YRD regional integration process to verify. The process of integration of YRD is mainly carried out from many aspects such as economic development, political system, and transportation facilities. From the perspective of the political system, the first joint meeting of mayors of cities in YRD was held in 1997, which opened the prelude of the cooperation between cities in the YRD region. In 2003, the fourth Yangtze River Delta Mayors' Summit was held, during which the concept of “People from the YRD” was first proposed, and a 16-city framework structure that has been stable and widely recognized for a long time has been established. In 2010, The Chinese State Council approved the implementation of the Regional Plan for the Yangtze River Delta, which expanded the original 16 cities to 25 for the first time at the national strategic level and explicitly proposed the construction of world-class city clusters with strong international competitiveness. In 2018, General Secretary Xi Jinping proposed to support the integrated development of the Yangtze River Delta region and make it a national strategy. In 2019, The Chinese State Council issued the Outline of the Yangtze River Delta Regional Integration Development Plan, which expanded the number of central cities to 27.

In terms of transportation facilities, by the end of 2019, the length of high-speed rail in operation in the YRD region had reached 4,997 km, forming a 1 to 3-h urban circle with Shanghai as the center, connecting Hangzhou, Nanjing, and Suzhou, Wuxi, Changzhou and Jiaxing in 1 h, Ningbo, Jinhua in 2 h and 3 h to Wenzhou, Hefei, Wuhu, and other cities. To sum up, the integration process of the YRD should be regarded as not only the process of changing institutional proximity, but also the process of changing organizational, social, cognitive, and even cultural proximity. In all, it is logically understandable that the influencing factors, which are endogenous variables of the model, are affected differently.

## Conclusions, implications, and limitations

STI is a concept with abundant meaning and is easily confusing. This study divides it into scientific innovation and technological innovation. According to the theory of multidimensional proximity and the division of knowledge type, we put forward our research hypothesis by their corresponding relations. Using the spatial panel econometric model, this study analyzes and discusses the spatial-temporal evolution of the SI level and TI level and their influencing factors with a research sample of 2001-2018 in China's YRD region, thus making a marginal contribution to bridging the knowledge gap in this field. The main conclusions of this study are as follows:

(1) Empirical results in the YRD region show that hypothesis (1) can be verified. Among the exogenous variables, geographical proximity has little influence on the SI level in the YRD region. The TI level is greatly affected by geographical proximity, that is, there is a significant spatial effect of geographical space on the TI level. This conclusion is essentially consistent with previous research conclusions on multidimensional proximity (Hansen, 2015; Davids and Frenken, 2018).
(2) Part of hypothesis (2) has been confirmed. Among the endogenous variables, the Economic development level, the Degree of opening up, and the Expenditure in S&T have a significant positive impact on the SI level in the YRD, while the Regional economic structure has a significant negative impact on the SI level in the YRD. Expenditure in education has no significant impact. The impact of the economic development level and regional economic structure on the TI level in YRD is significantly positive. The impact of the other three factors is not significant. No matter whether it is the SI level or TI level, the significant influencing factors all have spatial effects, that is, the explanatory variables in the neighboring area all affect the local dependent variables.
(3) The non-significant influencing factors and the influencing factors contrary to the coefficient sign are likely to be affected by proximity from other dimensions except geography. That is, endogenous variables are influenced by exogenous variables and change their significance and direction of influence. In this regard, Knoben and Oerlemans (2006) pointed out that the importance of geographical, organizational, and technological proximity should not be ignored, Yu and Yuizono (2021) also revealed that the impact of proximity in different dimensions on innovation performance varies in different regions. So the above conclusions need to be further refined and in-depth.

According to the above conclusions, this study draws the following theoretical implications:

It is necessary and capable to distinguish STIs, and this study tries to do so. That is, through the corresponding relationship between knowledge types and SI and TI, the total amounts of scientific literature and patents are regarded as the representational variables of SI and TI, which are used to measure the level of STI in specific regions. Accordingly, the influencing factors of SI and TI should also be dealt according to different regions, and this study explores from the perspective of multidimensional proximity, the proximity of different dimensions is regarded as the exogenous variable affecting the STI level according to its interaction mode, which proves that the endogenous variable affecting the STI level will change under the effect of multidimensional proximity as the exogenous variable. And this research paradigm, which considers both exogenous and endogenous variables, deserves further exploration.

For practical implications, the regional integration of the YRD should focus on the coordination of relevant innovation policies, avoid conflicts and competition among different cities' policies, and cultivate a social and cultural atmosphere conducive to STI. Consistent or close policy environment, social environment, and cultural environment are more conducive to the performance of regional innovation entities in STI. Specifically, “siphoning” and “trickle-down” effects of influencing factors may still exist between cities. Through policy coordination, institutional proximity can be directly changed, social proximity and cultural proximity can be indirectly changed, and then it has an impact on the performance of SI and TI in various regions.

In addition, there are significant spatial differences in the knowledge types of SI and TI, as well as in the degree of multidimensional proximity and influencing factors. Local innovation policies should be formulated considering the local conditions. Policies to encourage SI should be based on a global perspective, and links from other regions outside the YRD should be established based on multidimensional proximity. Policies to encourage TI can be considered more from the regional perspective, improve the geographical proximity by improving transportation accessibility, and undertake the spatial spillover of high-tech innovation regions. Meanwhile, policies to improve proximity from other dimensions should not be omitted to play a beneficial role in local TI.

The improvement of the regional STI levels is both a process and a result. Its characterization depends on the exploration from multiple theoretical perspectives. Based on the multidimensional proximity theory, this study classifies SI and TI based on knowledge types. The main conclusions are still based on the spatial panel model based on geographical proximity. For the spatial panel econometric model, the selection of spatial weight matrix depends on the specific research environment and has a very important influence on the analysis result. This study chooses the geographical spatial weight matrix may not fully explain the multidimensional proximity of the YRD region, namely the geographical boundaries of neighboring relationship may not fully reflect the YRD region of STI and its influencing factors, relationship based more on other dimensions is expected to further verify our research hypotheses. It still has enough research value consequently. In addition, the proximity of different dimensions may play different roles in different regions (Yu and Yuizono, 2021). Consequently, the proximity of different dimensions can also be considered as an independent variable to analyze the impact on the innovation level. Due to space limitations, this study cannot be expanded. Finally, since there may be a causal relationship between SI and TI, the simultaneous space equations model of the two can be considered for further studies.

## Data availability statement

Publicly available datasets were analyzed in this study. This data can be found here: www.cnki.net, www.webofscience.com, epub.cnipa.gov.cn.

## Author contributions

YY planned the study, collected, and analyzed the data and wrote the manuscript. JL improved the research design and research model. XH and YH co-analyzed the data. YY and JL were mainly involved in manuscript revision and editing. All authors listed have made a substantial, direct, and intellectual contribution to the work, and approved it for publication.

## Funding

This research was funded by National Natural Science Foundation of China (41976209); National Social Science Foundation of China (21BJL077); Humanities and Social Science Research Program of the Ministry of Education (21YJA630028); Zhejiang Provincial Soft Science Research Program (2022C35101); and Ningbo Soft Science Research Project (2021R027).

## Conflict of interest

The authors declare that the research was conducted in the absence of any commercial or financial relationships that could be construed as a potential conflict of interest.

## Publisher's note

All claims expressed in this article are solely those of the authors and do not necessarily represent those of their affiliated organizations, or those of the publisher, the editors and the reviewers. Any product that may be evaluated in this article, or claim that may be made by its manufacturer, is not guaranteed or endorsed by the publisher.

## Footnotes

^1^Of course, proximity affects innovation through more than just knowledge sharing. Studies have shown that it can also drive innovation by passing on the pressure of environmental regulation (Hu et al., 2021).

^2^They include the Yangtze River Delta, the Pearl River Delta, the Beijing-Tianjin-Hebei Region, the Middle Reaches of the Yangtze River, and the Chengdu-Chongqing urban agglomeration.

^3^ http://epub.cnipa.gov.cn/

^4^In this study, the spatial error model (SEM) is also used for the analysis, but λ is not significant, and the ρ≠-θβ in LR test, indicating that SEM is not applicable.

^5^Limited by space and subject matter, this article will not be discussed in detail.

^6^After we try to replace the spatial weight matrix with the second-order nearest neighbor (K2) and third-order nearest neighbor (K3) matrix, the magnitude and significance of the coefficient have changed. Limited by space, not listed here, available.
